# Informing the development of an online self-management program for men living with HIV: a needs assessment

**DOI:** 10.1186/1471-2458-14-1209

**Published:** 2014-11-24

**Authors:** Tanya Millard, Karalyn McDonald, Julian Elliott, Sean Slavin, Sally Rowell, Sonya Girdler

**Affiliations:** Department of Infectious Diseases, Monash University, Melbourne, Australia; Australian Research Centre in Sex, Health and Society, La Trobe University, Melbourne, Australia; Infectious Diseases Unit, Alfred Hospital, Melbourne, Australia; Burnet Institute, Melbourne, Australia; Centre for Social Research in Health, University of New South Wales, Sydney, Australia; Hepatitis WA, Perth, Australia; School of Occupational Therapy and Social Work, Centre for Research into Disability and Society, Curtin University, Perth, Australia

**Keywords:** HIV/AIDS, Men, Quality of life, Psychosocial issues, Self-management, Needs assessment, Positive Outlook

## Abstract

**Background:**

The aim of this mixed methods study was to conduct a multifaceted needs assessment to inform the development of an online self-management program for men living with HIV. The objectives were to describe the health-related quality of life for men living with HIV, the impact of living with HIV, and the perceived problem areas and service and support needs of these men. The needs assessment was conducted in accordance with the PRECEDE model for health promotion program planning.

**Methods:**

A survey assessing the quality of life of men living with HIV (n = 72) was conducted and results were compared to Australian normative data. Focus groups were also undertaken with men living with HIV (n = 11) and a multidisciplinary team of service providers working in the area of HIV (n = 11). Focus groups enabled an in-depth description of the impact of HIV on quality of life and perceived problem areas in daily life.

**Results:**

HIV-positive men experience significantly lower quality of life when compared with Australian normative data, particularly in those domains concerned with social and emotional aspects of quality of life. Qualitative focus groups yielded an overarching theme ‘The psychosocial impact of HIV’ which contained three sub-themes; (1) Life before and after HIV – a changed identity and its repercussions; (2) Resilience and the importance of social support; (3) Negotiating the practicalities – intimate relationships and disclosure.

**Conclusions:**

The findings from this needs assessment highlight the need to target socio-emotional contexts of HIV positive men’s daily lives to improve quality of life and well-being. Intervention priorities for the proposed online self-management program include: (1) managing the emotional impact of HIV; (2) disclosing HIV status to family and friends; (3) maintaining social connectedness; (4) managing HIV within intimate relationships; and (5) disclosure of HIV status to intimate partners.

## Background

Over the past decade in Australia, there has been an increase in the rate of new diagnoses of Human Immunodeficiency Virus (HIV) infection. This increased incidence, coupled with an aging population due in large part to the success of combination anti-retroviral treatment (cART), means that the number of people living with HIV (PLHIV) is projected to increase considerably in the future [1]. As HIV has developed into a chronic condition, it is important to identify the type of support and education needs of different populations presenting with HIV and target interventions accordingly. The shift to a chronic condition model of care in the population has resulted in recommendations for a self-management approach to be adopted [2–4].

Self-management involves three tasks: medical management, role management and emotional management, and encompasses six core skills: problem solving, decision making, resource utilisation, the formation of a patient-provider partnership, action planning and self-tailoring [5]. In order to be a successful self-manager, motivation, healthy behaviours and effective collaboration with health professionals is required [6]. Self-management interventions are widely used and have demonstrated effectiveness in improving health related outcomes and quality of life (QOL) for a variety of chronic conditions including diabetes [7], arthritis [8], vision loss [9], and cardiovascular disease [10]. Benefits to be obtained for people living with chronic conditions participating in self-management interventions, include reduced morbidity, improved health related behaviours and health outcomes, reductions in use of acute medical services and hospital visits and improved quality of life [11].

Self-management interventions have also been trialled among PLHIV with systematic reviews finding evidence of effectiveness enhancing adherence to cART [12], increasing exercise [13, 14], and short-term improvements in physical, psychosocial and health knowledge and behavioural outcomes [4]. In comparing common factors across chronic diseases and challenges which are specific to HIV, Swendeman and colleagues (2009) report “lack of direct self monitoring of physical status; stigma and disclosure; and criminalisation of HIV exposure” as particularly challenging when it comes to the self-management of HIV infection [15].

The development of well-informed, effective health interventions is a priority for health care providers targeting chronic conditions. Concern has been raised, however, that health education has focused too narrowly on the implementation of programs, rather than on designing and developing programs that are grounded in theory and systematically targeted towards meeting the concerns of the specific client groups [16, 17].

Undertaking a needs assessment has been established as an integral component in underpinning the development of effective health interventions including self-management programs [18]. Needs assessments systematically identify relevant information to determine action plans for commissioning new health interventions [18, 19]. Needs assessment for health problems evaluate QOL and the health status of a target population and identify factors influencing health [20]. The physiological, behavioural and environmental factors influencing the health problem are analysed and a list of priorities developed [20].

The aim of this study was to conduct a multi-faceted needs assessment in order to inform the development of an online self-management program for men living with HIV. The objectives of the needs assessment were to describe the health related QOL, impact of living with HIV and perceived problem areas in daily life for HIV-positive men. The needs assessment was conducted in accordance to the PRECEDE model for health promotion program planning [17].

### The PRECEDE model

The PRECEDE model provides a structure for the systematic application of theories and concepts for planning and evaluating health education and promotion programs [21]. The acronym PRECEDE stands for Predisposing, Reinforcing, and Enabling Constructs in Educational/Ecological Diagnosis and Evaluation [17]. The structure provided by the PRECEDE model enhanced the understanding of the impact of HIV, allowing us to assess the QOL of the men living with HIV in Australia and to identify the behavioural and environmental factors impacting QOL and priority areas for the men. The PRECEDE model promotes the use of numerous and varied information sources [20]. Accordingly, a multi-faceted needs assessment using both qualitative and quantitative methodologies was used to provide a comprehensive understanding of the impact of HIV on the lives of HIV-positive men in Australia.

The needs assessment consisted of four steps. First, the literature examining the epidemiology and impact of HIV amongst men was reviewed. Second, a survey assessing QOL of men living with HIV was conducted and results were compared to Australian normative data. The third step involved focus groups to obtain a more in-depth description of the impact of HIV on QOL and perceived problem areas in daily life. A focus group was also conducted with a multidisciplinary team of service providers working in the area of HIV to explore their perspective on the impact of HIV and identify problem areas. Finally, a systematic literature review was conducted, which examined the effectiveness of existing HIV-specific self-management interventions and identified those programs and models which could be used to inform the intervention targeting the needs of this group. Briefly, this review found that self-management programs for PLHIV result in short-term improvements in physical, psychosocial and health knowledge and behavioural outcomes [4]. The long-term effectiveness of such programs could not be established due to insufficient evidence. The present paper reports on the findings of the survey of quality of life and the focus group discussions.

## Methods

Participants for this study were recruited through a variety of HIV services across Perth, the capital city of Western Australia, including hospitals, clinics and community organisations. Participants were eligible for inclusion if they were HIV-positive, male, over the age of 18, and could read and write in English. Flyers advertising the study were displayed in waiting rooms and doctors, nurses and staff also assisted recruitment by identifying eligible participants and requesting their participation. Eligible participants were provided with a recruitment pack containing information and consent forms, a demographics survey and quality of life questionnaire. In order to increase recruitment and reduce the responsibility placed on health care providers, recruitment packs were also located in waiting rooms and reception areas so potential participants could take them at will. Due to this, there was no way to track the number of recruitment packs distributed and thus, response rate calculations were not possible. Recruitment packs were distributed between January and June 2011 and included a stamped, addressed envelope to return completed surveys.

The SF-12® was selected to measure health-related QOL. The survey consists of eight subscales including: physical functioning, role limitations due to physical health problems, bodily pain, social functioning, general mental health, role limitations due to emotional problems, vitality and general health perceptions [22]. The psychometric properties have been tested and demonstrated to be sound amongst a variety of general and disease specific populations (reliability coefficient median 0.76; relative validity median = 0.97) [22]. It is also considered a valid measure for use with PLHIV [23].

In addition to completing the survey, the information and consent forms invited participants to provide their contact details if they were willing to participate in a focus group discussion. The researcher then contacted participants who indicated interest and invited them to attend a focus group discussion. Focus groups were conducted to obtain a more in-depth description of the impact of HIV on QOL and identify the specific intervention priorities for the proposed self-management program. Naturalistic inquiry was selected as the theoretical perspective as it focuses on human behaviour in everyday life [24]. The groups were facilitated by the primary researcher (TM) and held at the Western Australian AIDS Council (WAAC). A discussion guide comprising 12 open-ended questions exploring the impact and experience of living with HIV was used to guide the focus groups. Discussions were audio recorded and transcribed verbatim. Personally identifying data was removed from the transcripts and pseudonyms have been used in the presentation of the findings. Due to time constraints and difficulty recruiting participants, saturation was not reached.

Convenience sampling was also used to recruit staff from various HIV support services, hospitals and community services supporting PLHIV in Perth to participate in a service provider focus group. The focus group explored physical, emotional and social issues experienced by men living with HIV, which could be addressed in the proposed online intervention. An interview schedule was used to guide the focus groups and ensure all areas were explored. This study was approved by the Human Research Ethics Committee at Edith Cowan University. All participants provided written consent to participate in this study.

### Data analysis

For the QOL survey, data was scored and transformed using the SF software resulting in the eight SF-12® health domains. Domain scores were exported to SPSS for analysis and compared with Australian normative data using one-sample t-tests [25]. It should be noted that normative data from the Australian population was collected using the SF-36®. Demographic and clinical characteristics of the sample were described using descriptive statistics.

NVIVO (a qualitative software package) was used as a data management tool for the qualitative component of the study. Thematic analysis identified repeated patterns of meaning within the focus group data [26]. The process of analysis involved the primary researcher (TM) reading the transcripts several times and coding the entire data set. Once data sets were coded they were organised into common themes [26]. Themes were discussed amongst the authors and further refined until consensus was met. Quality of the research method was assured by the authors’ comprehensive awareness of the relevant literature and competence and skills in qualitative research and thematic analysis.

## Results

### Survey of quality of life

A total of 72 participants returned completed surveys. Due to the sampling method, response rate calculations were not possible. The average age of participants was 50.78 years (SD = 10.92; median = 47) with a range of 27–82. The majority of the men identified as MSM (94%), with only four men identifying as heterosexual. Nearly a third were either married or defacto, 60% reported never being married/defacto and 10% reported being widowed/divorced. Over half of the men (54.2%) held a tertiary qualification, one third had completed secondary school as their highest level of education and 4% reported having completed only primary school or less. More than one third (42%) of the participants were in full time employment, 15% reported part time employment, 4% casual/seasonal work and 38% were unemployed or retired.

The average year of HIV diagnosis was 1995 (SD = 7.06; range 1984–2010) and the average year participants began taking cART was 2000 (SD = 5.22; range 1988–2010). The majority of participants (87.5%) reported taking cART and approximately half (52.8%) reported having a co-existing condition. The most frequently reported co-existing conditions included depression (19), followed by hypertension (6), hepatitis C (5), hepatitis B (5), high cholesterol (4), peripheral neuropathy (3) and diabetes (3).

As outlined in Table 1, participants scored significantly lower than the Australian norms for males on six of the eight domains of the SF-12® including physical functioning (p < 0.03), role physical (p < 0.02), vitality (p < 0.01), social functioning (p < 0.01), role emotional (p < 0.01) and mental health (p < 0.01). The mental component summary score (MCS) was also significantly lower for participants (p < 0.01). We also compared mean scores for participants with Australian norms for all persons (including males and females). Comparisons revealed that the participants scored significantly lower than Australian normative population for all persons on four of the eight domains, including vitality (p < 0.01), social functioning (p < 0.01), role emotional (p < 0.01) and mental health (p < 0.01). MCS was again significantly lower for the participants when compared to Australian norms for all persons (p < 0.01).

**Table 1:** **SF12 scores compared to Australian norms for males and all persons**

SF-12 health domain	Std. error	Participant mean scores	Std. error	Participant mean norm based score	Australian norms for males P-value *a*	Australian norms for all persons P-value *a*
Physical Functioning (PF)	3.77	76.05	1.29	48.24	0.034*	0.87
Role Physical (RF)	3.58	72.61	1.32	47.08	0.022*	0.46*
Bodily Pain (BP)	3.66	76.81	1.49	47.99	0.75	0.997
General Health (GH)	3.56	65.55	1.53	47.14	0.111	0.094
Vitality (V)	3.13	54.22	1.26	49.45	0.001***	0.002***
Social Functioning (SF)	3.99	64.79	1.61	42.35	0.001***	0.001***
Role Emotional (RE)	3.33	71.14	1.49	43.17	0.001***	0.001***
Mental Health (MH)	2.47	61.79	1.20	45.19	0.001***	0.001***
Physical Component Summary (PCS)	1.49	49.52	1.49	49.52	0.700	0.854
Mental Component Summary (MCS)	1.43	44.00	1.44	44.00	0.001***	0.001***

Higher scores signify better health. *a =* One-sample T-Test. *p < 0.05. ***p < 0.001.

### Focus groups

A total of 11 men participated in three focus groups. Participants were aged between 32 and 68 years (M = 52, SD = 12.48) and had been diagnosed between 1984 and 2009 (M = 1996, SD = 7.75). All participants identified as gay or MSM. Focus groups went for an average duration of 90 minutes. Two main themes arose from the focus groups, the psychosocial impact of HIV and frustration with current services and supports. For the purposes of this paper, we report on the main theme, the psychosocial impact. It is beyond the scope of this paper, and the study as a whole, to address participants’ issues with currently available services and supports. Additionally, because this study was conducted amongst a sample of men living in Western Australia, findings would not be representative of the services and supports offered in other states and territories.

A total of 11 people attended the service provider’s focus group. For the purposes of this paper, we focus only on the content discussing psychosocial issues. The issues presented in this paper are those they believed were having the largest impact on the lives of positive men and currently neglected by available supports and services. In the following results section, we present the findings of the participant and service provider focus groups together.

### The psychosocial impact of HIV

The substantial impact of HIV on psychosocial areas of participant’s lives dominated the men’s descriptions of their experience of living with HIV. The men clearly identified that a diagnosis of HIV no longer represented a death sentence and that it was generally manageable, at least on a physical basis, by adhering to their medication regime. However, they described psychosocial issues as those with which they continued to struggle: *“Apart from having to knock back a pill or attend your doctor’s on a regular basis this illness has little physical reality, it’s all psychosocial. And that’s sort of always there.”*

The prominence of psychosocial issues impacting the daily lives of men living with HIV was echoed in the service provider focus group:

*The psychological stuff is the stuff that really matters. The physical stuff can be largely managed with medications, but it’s the psychological issues that, from the work I’ve done, is probably having the greatest impact on people. The sort of fear of what’s going to happen to me later on, fear of what happens if you tell people. Because there is so much misunderstanding you know.*

Overall, the service providers reiterated the importance of addressing the psychosocial aspects associated with HIV in the proposed self-management intervention.

Patterns in the data revealed that the overarching theme of the psychosocial impact of HIV contained three sub-themes; (1) Life before and after HIV – a changed identity and its repercussions; (2) Resilience and the importance of social support; (3) Negotiating the practicalities – intimate relationships and disclosure. Theoretical thematic analysis was applied to the data as to identify these sub-themes. This allowed the researchers to focus on the overarching ‘psychosocial’ theme and provide a detailed analysis of this theme [26]. Pseudonyms are used in the following presentation of findings.

### Life before and after HIV- a changed identity and its repercussions

For many of the participants, receiving a HIV-positive diagnosis was described as a life-changing event. They recounted feeling as though they had to alter and adapt due to the impact HIV had on numerous areas of their lives. This theme was reiterated in the service provider focus group highlighted here by one participant:

*A diagnosis of HIV changes the way a person thinks and feels about themself, so their self-identity. I have worked with a lot of people to learn how to incorporate HIV into their identity without letting it overtake their identity. Some people make the mistake initially of thinking ‘I am a HIV-positive person, everything is about HIV,’ whereas they have to learn to be a person who has HIV, not, HIV is them.*

The sense of going through an identity transition, from the person they were prior to HIV, to their new self, living with HIV was articulated by Damian: “*But coming to terms with the difference between your sex life and your social life as well, I mean, Wow… You really do feel like you’ve been amputated from your life and you have to come up with a new identity in a way.”* Damian’s use of the term “amputation” indicated he no longer identified with the person he was prior to his diagnosis and that everything he did was now going to be impacted in some way or another by HIV.

Two other men gave similar accounts, highlighting how a changed sense of identity can result in withdrawal: *“Now I’m basically just quiet, I’ve sort of settled into a quiet existence,” “I became much more reclusive, and introverted”.* For these men, HIV had a profound impact on the way they lived their lives. The significant changes associated with their diagnoses resulted in a shift in priorities, and often, their willingness to participate in previously enjoyed occupations and roles.

The men provided numerous examples of the ways in which HIV had made them more withdrawn and selective within friendships. A prominent sense of loneliness emerged throughout the focus groups and may be considered a consequence of this changed sense of identity and the resulting withdrawal. Some of the men described losing the desire to go out and ‘party’ as they used to. Anthony explained that his decision not to seek intimate relationships following his positive diagnosis came with consequences: *“It’s very difficult to have friends who have families or kids or partners, whatever, if you’re not in that world. It’s very difficult. I suppose you’d have to say that it’s a lonely world.”*

The men provided examples of stigma expressed by their family, friends, existing and potential partners. This subtle withdrawal appeared at times to be linked with perceptions of stigma, compounding difficulties pursuing and maintaining friendships and relationships highlighted by Paul:

*Well I don’t know whether it’s because people exclude you or not, but yeah you do have less friends. And I don’t know if maybe it’s because of trust, you know you put up barriers. So that group narrows.*

Similarly, Damian’s perceptions of stigma contributed to the overall isolating impact of HIV in his life.

*Yeah, well, I want to go out and boogie (laughs) but I go out and look at my friends or other people and I think well I’m not associating with your cause you’re a bigot and I’ve had trouble with that person and yeah it does feel for me very strongly HIV-related.*

Stigma within the gay community was also discussed, with the men agreeing that it is a significant problem often compounding the isolating impact of HIV:

*There is a lot of nonsense talked about how it’s brought the gay community together… There’s kind of almost a snobbery amongst those who believe themselves to be negative, oh I’m negative because I’ve been careful. This is where the word clean comes in… As opposed to dirty you know. And you think they would be understanding being gay but they really aren’t. It isn’t the case at all. Often they are actually the worst.*

For men living with HIV, the subtle withdrawal from previously enjoyed occupations and roles and the potential impact this can have on other areas of life, and ultimately well being, requires more attention. Finding ways to assist positive men adjust to their diagnoses without sacrificing their sense of self, and disengaging from their social engagements, is an important concern for programs and support services targeting this population. An emphasis on dealing with stigma, maintaining social connectedness and seeking support should be a priority for services targeting the needs of men living with HIV.

### Resilience

Although all of the men described an overwhelming sense of shock when first diagnosed with HIV, participants repeatedly highlighted their belief that focusing on only the negative aspects of being HIV-positive was “*not an option*” for themselves or others. Participants held the belief that people who did this became *“possessed and obsessed”* with their diagnoses and health. The men clearly described making a choice to *“get up and get on with it”.*

*It sort of changed my life in that I went through a sort of transition where I went from being really depressed and down about it and then I sort of thought well stuff this, it’s either I’ll get up and get on with it or you just give in. That’s what you have to get into your head that you are not dying from it, you are living with it. And that’s what you should continue to do.*

Collectively, the men identified the importance of social support in adjusting to their positive diagnosis. They recounted seeking out other HIV-positive people at the time of their diagnosis and the ensuing sense of relief they felt being able to talk with someone who had been through a similar experience:

*I only knew one positive person and I found myself seeking him out and going around to talk on a number of occasions. And I felt at the time that I was imposing… I’m sure he probably knows quite a few people and had encountered that previously. But I really needed someone to talk to, even if it was about superficial issues. It was the ability to sit and talk and to air your thoughts and worries without any paranoia going around you. Having someone to go to talk to is invaluable.*

The men also discussed the importance of having people in their social circle who were aware of their positive status and could provide support accordingly, in addition to those who they can actually talk openly and honestly to:

*Yeah, you have a few good confidants who understand the situation…And of course you have your family and they are a fairly strong support for me. Not that we really discuss it a lot but they are aware of it and they are supportive.*

Developing skills to enhance resilience and to support social connectedness emerged as the men found important to be incorporated into any intervention targeting PLHIV. Clearly, this should be an area of priority for the proposed self-management intervention.

### Difficulties negotiating the practicalities – disclosure and intimate relationships

The participants viewed disclosure of HIV status as a constant point of stress and uncertainty. All of the men described being cautious and highly selective when it came to disclosing their positive status, with the potential for rejection a prominent concern. When asked about the most difficult aspects of living with HIV, the men responded:

*Mark: Who do you tell and do you tell at all?*

*Allan: Yeah, how to tell and how to deal with the horrible reactions*

*Adam: Yeah I agree, dealing with rejection.*

Disclosure was viewed in very specific ways. The men described their strategies of evaluating the situation for disclosure. They reflected on the reasons why disclosure was needed, to whom to disclose and the predicted outcome of disclosure. One of the participants likened the process of disclosing positive status to the process of ‘coming out’ as a gay man:

*You know the coming out process when you’re gay there’s also a second coming out process when you’re HIV and to this day there are people who I will not disclose my HIV status to.*

The service providers also identified disclosure of HIV status, particularly within intimate relationships, as a widespread issue. Aspects including helping the men navigate whom to disclose, how to disclose, dealing with rejection and supporting those who they have disclosed to, were identified as important:

*Relationships are a huge issue for men, you know like how do you meet somebody, how do you disclose all of that, I think that’s pretty universal. It doesn’t matter how confident you are in yourself but I still think it’s a tough gig to have to disclose it to somebody.*

Collectively, participants described the detrimental impact HIV had on their sex lives and a sense of loss over missed sexual opportunity emerged. For many of the men, maintaining an active sex life was considered too complicated and thus, was an area of life they no longer pursued. When asked about the main adjustments he had made to his life since receiving his positive diagnosis, Peter identified those regarding sexual relationships. He described previously enjoying his sex life, however, the complications associated with HIV meant that he no longer sought physical intimacy: *“I’ve moved over time to not wanting relationships that are physically intimate because I’m just not interested any longer… I like an uncomplicated life*.”

The men discussed numerous reasons behind their decision to no longer pursue intimate relationships, including general difficulties seeking relationships, wishing to avoid disclosure, not wanting to put others at risk, and fear of rejection. Matt highlighted the multitude of complex issues PLHIV face when pursuing intimate relationships, saying “*I stopped having sex after sero-conversion, just couldn’t, wasn’t, wouldn’t”.* Thinking about Matt’s words, “couldn’t, wasn’t, wouldn’t” provides us with deeper insight into many of the issues associated with sexual intimacy and HIV; the reasons he “couldn’t” (continue having sex following sero-conversion) may be attributed to fear of disclosure or performance issues as a consequence of emotional factors or medication side effects; when he says “wasn’t” he may be referring to lack of interest, willingness or ability; and finally “wouldn’t” may refer to his perceptions of putting others at risk, or risk rejection and the possibility that he will not pursue sexual intimacy anymore.

A couple of the men who were in relationships at the time of diagnosis described examples of rejection following disclosure of their positive status to their partners, highlighted in this extract from one of the focus groups:

*Peter: The guy I was seeing at the time, I told him I had HIV and he said, oh well, it’s over then… and he left.*

*Paul: Yeah, my partner hopped up off the bed and said, well I’m never coming around here again and walked out. And I was like hold on, isn’t there a conversation that needs to happen here?*

The issue of sexuality and managing intimate relationships also featured prominently in the service providers’ focus group. One of the biggest challenges identified by a group member was the *“grief of the missed sexual opportunity for the men.*” He described the importance of increasing men’s awareness and understanding of risk reduction strategies that may enable them to continue to have a satisfying sex life:

*How do they manage their sexuality now with HIV? How are they going to continue in the future to still have an enjoyable sex life but incorporate their HIV into that and minimise the risk of transmission to somebody else?*

The difficulties faced by participants in disclosing their status to potential sex partners were a significant barrier to their willingness to seek intimate relationships. Considering the prominent and ongoing impact an HIV-positive diagnosis has on intimate relationships, and the multitude of barriers these men have cited preventing them from seeking and maintaining an enjoyable sex life, it is clear that more work needs to be targeted towards addressing this issue.

## Discussion

The purpose of the needs assessment was to establish priority areas to inform the development of an online self-management program for gay men living with HIV. The findings from the QOL survey highlighted the need to address psychosocial issues in the management of HIV. The five domains, role physical, vitality, social functioning, role emotional and mental health consistently demonstrated statistical significance. The mental component summary was significantly lower for participants in both comparisons (all persons and males). These findings were echoed in the focus groups, which highlighted the pervasive and significant impact of HIV and its prominent and ongoing psychological and social rather than physical impact. It is important to consider HIV not only as a physical event, but also as a psychological shift. For many people, the changes and adjustments required following a positive diagnosis have a sustained impact on many areas of their lives.

One of the main themes identified in the focus groups was the identity transition people with HIV often experience and the broad subtle impact this transition can have on a multitude of areas within the person’s life. The men acknowledge that the physical/medical aspects of HIV are largely manageable with cART, a position supported and promoted by the medical profession. However, the psychosocial and emotional issues resulting from an HIV diagnosis and the changes required or otherwise resulting, often profoundly impacts men’s identities and social engagement. PLHIV are, in many ways, living with a contradiction. They are told that HIV is now a chronic manageable condition considered in much the same way as diabetes, however, their diagnosis presents unique issues such as stigma and difficulties negotiating disclosure and intimate relationships, which continue to impact multiple areas of their lives [27, 28]. Whilst community organisations are becoming increasingly aware of this, more work is required in order to ensure PLHIV are getting the support they require to address these ongoing issues. The proposed self-management intervention could be a useful method of addressing this pressing issue.

The survey of quality of life indicated that HIV-positive men experience lower quality of life than the general population on the social functioning, role physical, vitality and role emotional domains. These findings were further explored in the focus groups where participants commonly described withdrawing from previously enjoyed occupations and social relationships. This withdrawal was not something that appeared to happen immediately post diagnosis, rather something that developed over time. Many of the men tried pursuing relationships or disclosing their positive status to others, however, often experienced rejection or stigma as a result. Alternatively, the fear of such responses was enough to prevent them even contemplating disclosure or seeking intimate relationships. Stigma, loneliness and social withdrawal appeared to feed into each other in a vicious cycle characterised by fear and avoidance. This finding is supported in research conducted by Webel and colleagues (2014) who found that HIV-related stigma is a significant predictor of stress and social isolation [29]. Despite education and initiatives by the communities supporting PLHIV in addressing the issues of stigma, it continues to have a strong and negative impact on the lives of positive men.

Consistent with previous research, disclosure emerged as a prominent issue for focus group participants [30–32]. In fact, participants identified disclosure as “the most difficult aspect of living with HIV”. The decision of to whom, when, where, and exactly how to disclose were factors cited as impacting their approach to disclosure. The barriers and facilitators to disclosure within intimate relationships are similar to those cited by Rutledge [33]. This finding is also consistent with the most recent HIV Futures Survey, where more than half of those surveyed agreed with the statement “I am afraid of telling potential partners of my HIV status in case they reject me” [34]. Disclosure within intimate relationships is an area that would be beneficial to target in the proposed online intervention.

The difficulties the focus group participants described regarding intimate relationships is also a theme repeatedly discussed in the literature [27, 35, 36]. The men and service providers recounted the dramatic impact a positive diagnosis had on their sex lives and the multitude of adjustments required to negotiate intimate relationships. Disclosure, legalities, the potential for rejection, fear of transmission and general difficulties seeking relationships were highlighted as significant concerns. The prominence of issues surrounding seeking and maintaining intimate relationships amongst PLHIV was highlighted in a large scale nation-wide survey in Australia where over a third (36.3%) of participants agreed with the statement, “I have stopped having sex because of my HIV status” and two thirds of respondents also agreed with the statement, “few people would want a relationship with someone who has HIV” [34]. Despite the significance of issues relating to intimate relationships, it is not a topic often discussed by positive men in their appointments with their GP’s. This is concerning considering that for many men, their GP is the only person they speak to regarding HIV related issues. Negotiating intimate relationships is a primary concern for men living with HIV and would be a useful addition to any program addressing QOL for men living with HIV.

There are several limitations to this study. Firstly, this study included only those who identified as gay or MSM, meaning that the results from this study may not be applicable to other PLHIV including women, heterosexual men and people who are culturally and linguistically diverse. Second, the focus groups were conducted with a relatively small sample of participants and saturation was not reached in the timeframe available. The multifaceted approach to the needs assessment including a comprehensive review of literature addressed this limitation, ensuring the findings were representative of the population of gay/MSM living with HIV in Australia as a whole.

## Conclusions

This paper has described the health related QOL, impact of living with HIV and perceived problem areas in daily life for HIV-positive men. The needs assessment has highlighted that psychosocial issues are often the largest barriers to quality of life for men living with HIV. While the physical health needs of PLHIV in Australia are being relatively well managed, HIV-positive men continue to experience significantly reduced quality of life as issues such as disclosure, intimate relationships and social isolation are not being adequately addressed. In response to these findings, an online self-management program called “Positive Outlook” has been developed and evaluated using a randomised controlled trial in 2013. Based on the findings from the present study, priority areas for the online program included: (1) managing the emotional impact of HIV; (2) disclosing HIV status to family and friends; (3) maintaining social connectedness; (4) managing HIV within intimate relationships; and (5) disclosure of HIV status to intimate partners. The findings of this trial will be published.
